# Anatomic Distribution of Benign Ovarian Tumors in Perimenopausal and Postmenopausal Women

**DOI:** 10.7759/cureus.34059

**Published:** 2023-01-22

**Authors:** Michail Matalliotakis, Charoula Matalliotaki, Konstantinos Krithinakis, Aggelos Laliotis, Georgios Kapetanios, Ioannis Tsakiridis, Ioannis Kalogiannidis

**Affiliations:** 1 Department of Obstetrics and Gynecology, Venizeleio General Hospital, Heraklion, GRC; 2 Department of General Surgery, Venizeleio General Hospital, Heraklion, GRC; 3 3rd Department of Obstetrics and Gynecology, Aristotle University of Thessaloniki, Thessaloniki, GRC

**Keywords:** postmenopause, perimenopause, benign ovarian tumors, adnexal mass, anatomic distribution

## Abstract

Introduction

We aim to report the histotypes and reassess the anatomic distribution of benign ovarian tumors in perimenopausal and postmenopausal women.

Methods

Medical and pathology reports of women with histologically confirmed benign ovarian pathology were investigated. Data were collected, retrospectively between 2000 and 2020, and analyzed from perimenopausal and postmenopausal women with benign ovarian tumors, after bilateral salpingo-oophorectomy (BSO) with or without total abdominal hysterectomy (TAH). The ovarian masses histology and the distribution of locations were further evaluated.

Results

The total sample consisted of 1,355 women with benign ovarian tumors; 929 (68.6%) of the perimenopausal and 426 (31.4%) of the postmenopausal age. A dermoid cyst was prominent in the right ovary (52.8%), compared to the left side (41%) (p<0.01). Conversely, in patients with endometriomas and cysts of Morgagni, the observed proportion was more prominent in the left-sided ovary (61.8% vs 27%; p<0.001 and 52.3% vs 36.4%; p<0.01, respectively). Moreover, in the perimenopausal women, we mostly detected endometrioma (18.3%), dermoid cyst (15.5%) and cyst of Morgagni (4%) compared to postmenopausal women, where serous cysts (29.8%) and ovarian fibroids (8%) were the most common tumors.

Conclusions

Benign ovarian tumors are frequently seen in perimenopausal women and most histotypes present anatomical differences between the left and right ovaries. Serous cysts, followed by paraovarian, dermoid cysts and endometrioma present the commonest ovarian benign masses. Gynecologists should pay special attention to adnexal tumors in the postmenopausal period to choose the right operating setting for women at risk for ovarian cancer.

## Introduction

Benign tumors of the adnexa can be separated into non-neoplastic and neoplastic lesions. Moreover, neoplastic lesions can be of physiologic or pathologic growth [1, 2]. Surface epithelial-stromal tumors, sex cord-stromal tumors, and germ cell tumors are the three main types of ovarian tumors [1]. Benign serous tumors present common lesions and are a part of the surface epithelial-stromal tumor with a tendency to occur bilaterally (20% of cases); they are typically thin-walled and unilocular [2,3].

Due to the absence of consistent reporting and further due to the spontaneous resolution of ovarian cysts, especially in the reproductive period, the epidemiology of ovarian cysts is not clearly reported. According to Dorum et al., the incidence of ovarian cysts varies with patient demographics and ranges from 5% to 15% [4]. Moreover, Whiteman et al. reported that 7% of gynecologic hospitalizations were for benign ovarian tumors, in the United States [5]. Considering all ages with various histological types, in the majority of cases, ovarian masses are benign. On the other hand, the overall incidence of ovarian cancer increases with age up to the mid-seventies and declines among cases beyond 80 years [2, 6].

Several studies have investigated the topography and characteristics of benign and malignant ovarian tumors. In these studies, ovarian endometriomas are mostly reported [7-9]. Noteworthy, a left lateral predisposition of endometrioma and a right lateral predisposition of teratoma have been previously reported [10,11].

In the framework of this study, we aim to report the histotypes and delineate the anatomic distribution of benign ovarian tumors in perimenopausal and postmenopausal patients.

## Materials and methods

This retrospective study was carried out at the Department of Obstetrics and Gynecology at Venizeleio General Hospital of Heraklion Crete, between 2000 and 2020 and the Third Department of Obstetrics and Gynecology of Aristotle University of Thessaloniki, between 2009 and 2020. All women aged ≥40 years that underwent bilateral salpingo-oophorectomy (BSO) with or without total abdominal hysterectomy (TAH) and the final histology detected benign ovarian tumors were eligible to participate in the study.

Data were collected including age, menopausal status at surgery, side and macroscopic characterization of the ovarian cyst recorded at the operating theater. We excluded cases with incomplete medical records and those lacking histological evidence. Furthermore, patients with ovarian malignancy diagnosed upon histopathology were also excluded from the final analysis.

The clinicopathologic features of the ovarian tumors were classified according to the criteria of FIGO [12,13]. The pathological classification of the cysts includes: 1) serous, 2) mucinous, 3) dermoid, 4) paraovarian, 5) Morgagni, 6) endometrioma, 7) ovarian fibroids and 8) miscellaneous cysts.

All the participants consented for the anonymity of their data and the possible use for research purpose, while no incentives were provided. The Ethics Committee of both Departments approved the protocol (no. 124/17/2019, no.94/23-4-20).

Statistical analysis

Student t-test and X^2^ test were used for the comparison of the mean of various characteristics. The Mann-Whitney U test was performed if data were not distributed normally. The results are reported as mean ± SD or as percentages, where appropriate.

Differences were considered statically significant at p<0.05. Graphs were generated using GraphPad Prism (GraphPad Software, La Jolla, CA).

Data availability

The data associated with the paper are not publicly available but are available from the corresponding author on reasonable request.

## Results

Over a 20-year period, 1,355 women with benign ovarian tumors were treated and evaluated. Women were divided into group I, which included 929 patients in perimenopause, between 40 and 54 years old, and group II, which included 426 women in postmenopausal age between 55 and 84 years old. Histopathological characteristics of all cases studied are illustrated in Table 1.

**Table 1:** Characteristics of anatomic distribution of ovarian cysts in 1,355 women.

Diagnosis	No (%)	p-value
1. Serous cysts	n=274/1,355 (20.2%)	
Left-sided	111 (40.5%)	
Right-sided	116 (42.3%)	N.S.
Bilateral	47 (17.2%)	
2. Mucinous cysts	n=45/1,355 (3.3%)	
Left-sided	23 (46%)	
Right-sided	19 (42.3%)	N.S.
Bilateral	3 (6.7%)	
3. Dermoid cysts	n=178/1,355 (13.1%)	
Left-sided	73 (41%)	
Right-sided	94 (52.8%)	p<0.01
Bilateral	11 (6.2%)	
4. Paraovarian cysts	n=250/1,355 (18.5%)	
Left-sided	74 (29.6%)	
Right-sided	69 (27.6%)	N.S.
Bilateral	107 (42.8%)	
5. Cysts of Morgagni	n=44/1,355 (3.3%)	
Left-sided	23 (52.3%)	
Right-sided	16 (36.4%)	p<0.01
Bilateral	5 (11.4%)	
6. Endometrioma	n=178/1,355 (13.1%)	
Left-sided	110 (61.8%)	
Right-sided	48 (27%)	p<0.001
Bilateral	20 (11.2%)	
7. Ovarian fibroids	n=42/1,355 (3.1%)	
Left-sided	15 (35.7%)	
Right-sided	19 (45.2%)	N.S.
Bilateral	8 (19.1%)	
8. Miscellaneous cysts	n=344/1,355 (25.4%)	
Left-sided	107 (31.1%)	
Right-sided	110 (32%)	N.S.
Bilateral	127 (36.9%)	

**Figure 1:**
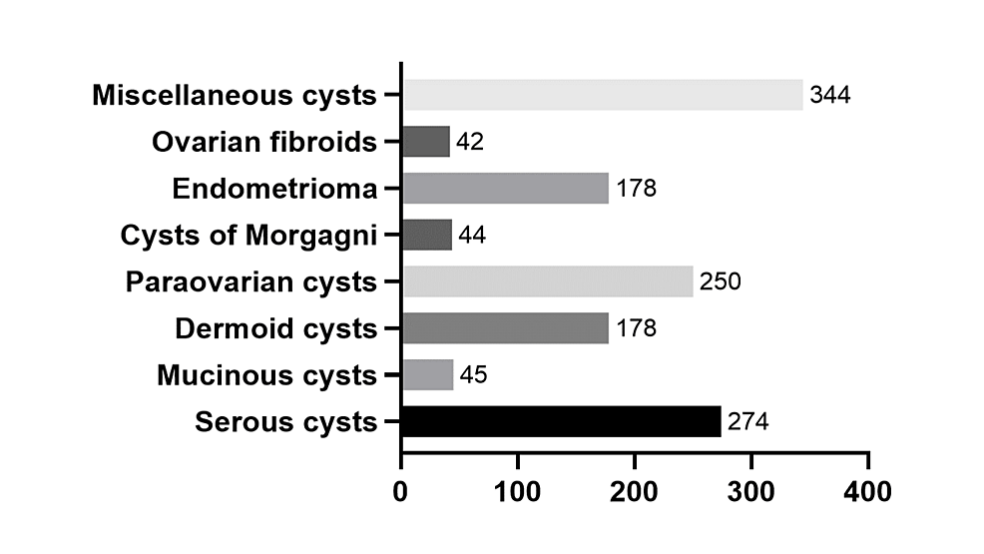
Histological distribution of benign ovarian tumors of our whole sample analysis.

In total, miscellaneous cysts were the most frequently diagnosed tumors (n=344; 25.4%), followed by serous (n=274; 20.2%), paraovarian (n=250; 18.5%), dermoid cysts (n=178; 13.1%) and endometriomas (n=178; 13.1%) (Figure 1).

**Figure 2:**
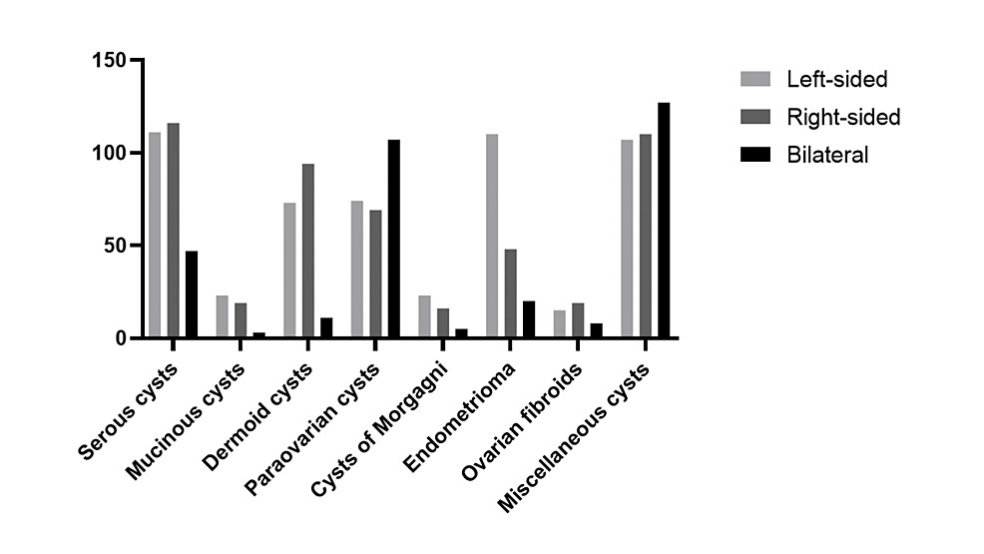
Anatomical distribution of ovarian masses (total sample).

The dermoid cysts seemed to develop more frequently in the right ovary (p<0.01). In contrast, cases with endometriomas and cysts of Morgagni were significantly more frequently detected in the left-side (p<0.001 and p<0.01, respectively). Figure 2 illustrates the anatomical distribution of ovarian masses.

With regards to the location of the tumors, no significant differences were detected in serous, mucinous, paraovarian, ovarian fibroids and miscellaneous cysts.

**Figure 3:**
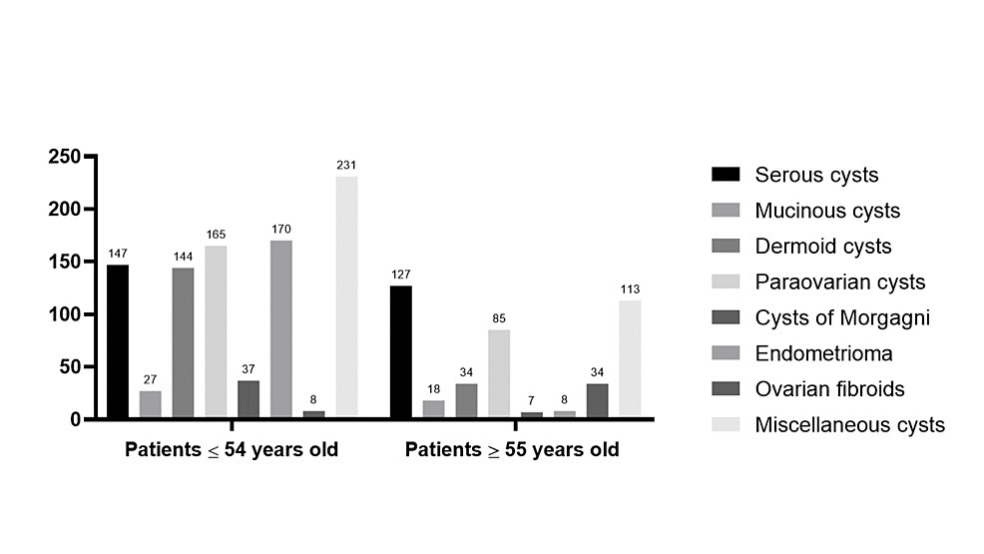
Age-related difference in distribution of ovarian masses.

Regarding the two age-groups, we mostly observed endometriomas (p<0.001), dermoid (p<0.01) and cysts of Morgagni (p<0.05), in the perimenopausal women, while in the postmenopausal group, we mostly detected serous cysts (p<0.01) and ovarian fibroids (p<0.001) (Figure 3).

The frequency of histotypes and the anatomical locations of the ovarian tumors in 929 perimenopausal and 426 postmenopausal women are shown in Table 2.

**Table 2:** Anatomic distribution of ovarian cysts in 929 perimenopausal and 426 postmenopausal women (N and %).

Diagnosis	Patients <55 years old, n=929 (68.6%)	Patients ≥55 years old, n=426 (31.4%)	p-value
1. Serous cysts n=274			
Left-sided n=111	63 (42.9%)	48 (37.8%)	
Right-sided n=116	70 (47.6%)	46 (36.2%)	
Bilateral n=47	14 (9.5%)	33 (26%)	
Total	147 (15.8%)	127 (29.8%)	p<0.01
2. Mucinous cysts n=45			
Left-sided n=23	13 (48.2%)	10 (55.6%)	
Right-sided n=19	14 (51.8%)	5 (27.8%)	
Bilateral n=3	0	3 (16.6%)	
Total	27 (2.9%)	18 (4.2%)	N.S.
3. Dermoid cysts n=178			
Left-sided n=73	59 (41%)	14 (41.2%)	
Right-sided n=94	76 (52.8%)	18 (53%)	
Bilateral n=11	9 (6.2%)	2 (5.8%)	
Total	144 (15.5%)	34 (8%)	p<0.01
4. Paraovarian cysts n=250			
Left-sided n=74	57 (34.5%)	17 (20%)	
Right-sided n=69	53 (32.1%)	16 (18.8%)	
Bilateral n=107	55 (33.4%)	52 (61.2%)	
Total	165 (17.8%)	85 (19.9%)	N.S.
5. Cysts of Morgagni n=44			
Left-sided n=23	20 (54%)	3 (42.8%)	
Right-sided n=16	14 (37.9%)	2 (28.6%)	
Bilateral n=5	3 (8.1%)	2 (28.6%)	
Total	37 (4%)	7 (1.6%)	p<0.05
6. Endometrioma n=178			
Left-sided n=110	105 (61.8%)	5 (62.5%)	
Right-sided n=48	46 (27%)	2 (25%)	
Bilateral n=20	19 (11.2%)	1 (12.5%)	
Total	170 (18.3%)	8 (1.9%)	p<0.001
7. Ovarian fibroids n=42			
Left-sided n=15	4 (50%)	11 (32.4%)	
Right-sided n=19	3 (37.5%)	16 (47.1%)	
Bilateral n=8	1 (12.5%)	7 (20.5%)	
Total	8 (0.9%)	34 (8%)	p<0.001
8. Miscellaneous cysts n=344			
Left-sided n=107	77 (33.3%)	30 (26.5%)	
Right-sided n=110	79 (34.2%)	31 (27.4%)	
Bilateral n=127	75 (32.5%)	52 (46.1%)	
Total	231 (24.9%)	113 (26.5%)	N.S.

## Discussion

According to our study including women that underwent surgery for a benign ovarian tumor, the prevalence of ovarian masses was 68.6% in perimenopausal and 31.4% in postmenopausal women. With regards to age, endometrioma was the most common tumor in perimenopausal women and serous cyst was the most prominent tumor in postmenopausal women.

With the increased use of ultrasound and computed tomography, there is an increasing number of “pelvic masses” being identified [14].

Several combined approaches of evaluating ovarian adnexal masses have been proposed in the literature. Elevation of the serum CA 19-9 and CA-125 biomarkers, menopausal status, ultrasonographic imaging and clinical findings present useful predictors to differentiate between benign and malignant ovarian masses [15-17]. In our sample, ovarian tumors were detected either as a result of gynecological screening, an examination performed for a supposed pelvic mass, or incidentally following examination for other medical conditions. In the United States, around 5-10% of women with an adnexal lesion are operated and about 13-21% of those cases are malignant [18]. The differential diagnosis of ovarian tumors (fluid-filled or solid) varies considerably according to the age of women. In particular, in the perimenopausal period, most neoplasms are benign, while ovarian tumors in postmenopausal women should be considered malignant until proven otherwise [6, 13].

Functional cysts, serous and mucinous cystadenomas are common benign ovarian tumors; one out of four cases can be endometriomas and 30% mature cystic teratomas. These findings suggest that ovarian tumors are mostly seen before and during the perimenopausal period and are usually benign [2,19-21]. We confirmed that serous cysts, paraovarian cysts, endometriomas and dermoid cysts are found more frequently in our study. Moreover, we detected unilateral dermoid cysts more frequently in the right ovary. This observation is consistent with previously published data [11]. Moreover, our findings are in agreement with Vercellini et al. who investigated the lateral distribution of non-endometriotic benign ovarian cysts [7]. With regards to endometrioma and cysts of Morgagni, we found them more frequently in the left ovary, which is also in accordance with the results of previous studies [9,11, 22-24]. Of note, Matalliotakis et al. have suggested the left-sided female varicocele theory for the ovarian endometriosis, which suggests the compression of the left renal vein as a causative mechanism [10]. The different topographical locations of the above benign lesions may suggest that the pathogenetic mechanism of those lesions is different [7]. As previously mentioned, Matalliotakis et al. described the female varicocele theory for endometriosis [10], thus future studies will be needed to evaluate various anatomical features of the pelvic area that may explain the right or left distribution with various mechanisms, such as alternations in blood flow, lymphatic drainage or genetic, inflammatory and local growth factors.

Regarding postmenopausal women, the incidence of an incidentally identified unilocular ovarian cyst varies between 3% and 17%, with the majority of them being shrunk automatically [25]. Of note, in our study, serous cysts presented the commonest benign ovarian tumor, and this is consistent with previously published data [4]. Even though, the risk of malignancy is around 1% in unilocular echo-free cysts [25], Guleria et al. observed that women presenting with a benign ovarian tumor are at elevated risk of developing malignancy in postmenopause [26].

The present study has certain limitations that are mainly intrinsic to the retrospective nature of the study. However, the study consisted of an analysis performed on a large number of cases in perimenopause and postmenopause and this possesses a major strength of the current work.

## Conclusions

We detected endometriomas, dermoid cysts and cysts of Morgagni, predominantly in the perimenopausal women, while in the postmenopausal group, we mostly detected serous cysts and ovarian fibroids. Moreover, we observed anatomical differences between the left and right ovaries.

The existence of a benign ovarian tumor in perimenopause and postmenopause is not a harmless lesion and when detected should be evaluated and managed accordingly.
